# Co-development and testing of an extended community pharmacy model of service delivery for managing osteoarthritis: protocol for a sequential, multi-methods study (PharmOA)

**DOI:** 10.1186/s12891-023-07105-2

**Published:** 2024-01-12

**Authors:** Opeyemi O. Babatunde, Elizabeth Cottrell, Simon White, Adrian Chudyk, Emma L. Healey, John Edwards, Elaine Nicholls, Nicola O’Brien, Adam Todd, Christine Walker, Colin Stanford, Tania Cork, Angela Long, Joanna Simkins, Christian D. Mallen, Krysia Dziedzic, Melanie A. Holden

**Affiliations:** 1 Keele University, Primary Care Centre Versus Arthritis, School of Medicine, Keele, Staffordshire ST5 5BG UK; 2 Impact Accelerator Unit, Keele University, Keele, Staffordshire ST5 5BG UK; 3 Wolstanton Medical Centre Newcastle-Under-Lyme, Newcastle-under-Lyme, ST5 8BN UK; 4 Keele University, School of Pharmacy and Bioengineering, Keele, Staffordshire UK; 5 Keele Clinical Trials Unit, Keele University, Keele, Staffordshire UK; 6 Department of Psychology, Northumbria University, Newcastle-Upon-Tyne, Tyne and Wear UK; 7 Newcastle University, School of Pharmacy, Newcastle-Upon-Tyne, Tyne and Wear UK; 8 NHS Shropshire Clinical Commissioning Group, Shrewsbury, Shropshire UK; 9 North Staffs and Stoke Local Pharmaceutical Committee, Stoke-On-Trent, Staffordshire UK

**Keywords:** Osteoarthritis, Community pharmacy, Primary care, Self-management

## Abstract

**Background:**

Osteoarthritis is a common, painful and disabling long-term condition. Delivery of high-quality guideline-informed osteoarthritis care that successfully promotes and maintains supported self-management is imperative. However, osteoarthritis care remains inconsistent, including under use of core non-pharmacological approaches of education, exercise and weight loss.

Community pharmacies are an accessible healthcare provider. United Kingdom government initiatives are promoting their involvement in a range of long-term conditions, including musculoskeletal conditions. It is not known what an enhanced community pharmacy role for osteoarthritis care should include, what support is needed to deliver such a role, and whether it would be feasible and acceptable to community pharmacy teams. In this (PharmOA) study, we aim to address these gaps, and co-design and test an evidence-based extended community pharmacy model of service delivery for managing osteoarthritis.

**Methods:**

Informed by the Theoretical Domains Framework, Normalisation Process Theory, and the Medical Research Council (MRC) framework for developing complex interventions, we will undertake a multi-methods study involving five phases:

1. Systematic review to summarise currently available evidence on community pharmacy roles in supporting adults with osteoarthritis and other chronic (non-cancer) pain.

2. Cross-sectional surveys and one-to-one qualitative interviews with patients, healthcare professionals and pharmacy staff to explore experiences of current, and potential extended community pharmacy roles, in delivering osteoarthritis care.

3. Stakeholder co-design to: a) agree on the extended role of community pharmacies in osteoarthritis care; b) develop a model of osteoarthritis care within which the extended roles could be delivered (PharmOA model of service delivery); and c) refine existing tools to support community pharmacies to deliver extended osteoarthritis care roles (PharmOA tools).

4. Feasibility study to explore the acceptability and feasibility of the PharmOA model of service delivery and PharmOA tools to community pharmacy teams.

5. Final stakeholder workshop to: a) finalise the PharmOA model of service delivery and PharmOA tools, and b) if applicable, prioritise recommendations for its wider future implementation.

**Discussion:**

This novel study paves the way to improving access to and availability of high-quality guideline-informed, consistent care for people with osteoarthritis from within community pharmacies.

## Background

Osteoarthritis is common, painful and disabling [1], often occurring alongside other long-term conditions (LTCs) including cardiovascular and pulmonary conditions, hypertension, and diabetes [2]. Core management approaches outlined by the National Institute for Health and Care Excellence (NICE) osteoarthritis guidelines [3] comprise information provision, exercise and weight control. The recommendations are based on evidence demonstrating reduced pain and improved function resulting from these approaches [3]. Improving physical activity and controlling weight can also improve outcomes for other common co-existing conditions. Therefore, developing ways to support the delivery of osteoarthritis recommendations to successfully promote and maintain supported self-management should have wide positive outcomes for people with osteoarthritis.

Available data show that most people with osteoarthritis consulting with healthcare professionals consult with their family doctor; around one sixth consult a physical therapist [4–7]. However, osteoarthritis care is inconsistent. Family doctors tend to underuse core non-pharmacological approaches and widely use pharmacological strategies [4, 8–10], and physical therapists offer exercise, and are well placed to provide weight-loss and analgesia advice, but there is equivocal evidence that they have the confidence to do so in current practice [11, 12]. Opportunities for promoting guideline-informed supported self-management for people with osteoarthritis are therefore currently being missed [13].

Prior, or in addition, to seeking formal medical care, people with LTCs such as osteoarthritis often seek support from community pharmacies [14], as they are readily accessible in local communities. Over 10,000 community pharmacies are available in England, although since 2015 this number has been declining [15]. UK policy recommendations to integrate pharmacists into LTC pathways and national workforce initiatives have promoted community pharmacy roles [16, 17]. Community pharmacist roles have extended over the last twenty years beyond just supplying medicines and managing medicines-related problems into more ‘cognitive services’ that includes medicines optimisation [17–21]. Their role is now recognised as “supporting people with LTCs to improve their quality of life, health and wellbeing and to lead as independent a life as possible by supporting self-care” [22]. Given current government initiatives to enhance contact with pharmacies, supporting community pharmacy services to deliver better care to people with osteoarthritis is logical and needed. Similar initiatives are currently being explored in other countries [23–25], however, the expected role of community pharmacies in delivering osteoarthritis care, necessary support or pathways to provide this care, and patient and public awareness of the breadth of community pharmacy roles are not well understood/established in the United Kingdom [20, 21]. Specifically, little is known about: what an enhanced community pharmacy role for osteoarthritis care should include, what support is needed to fulfil such a role, and whether it would be feasible to deliver and acceptable to community pharmacy teams.

We will address these gaps in knowledge, and in order to improve the availability of consistent guideline-informed care, we aim to co-design and test an evidence-based extended community pharmacy model of service delivery for managing osteoarthritis.

## Methods

**Fig. 1:**
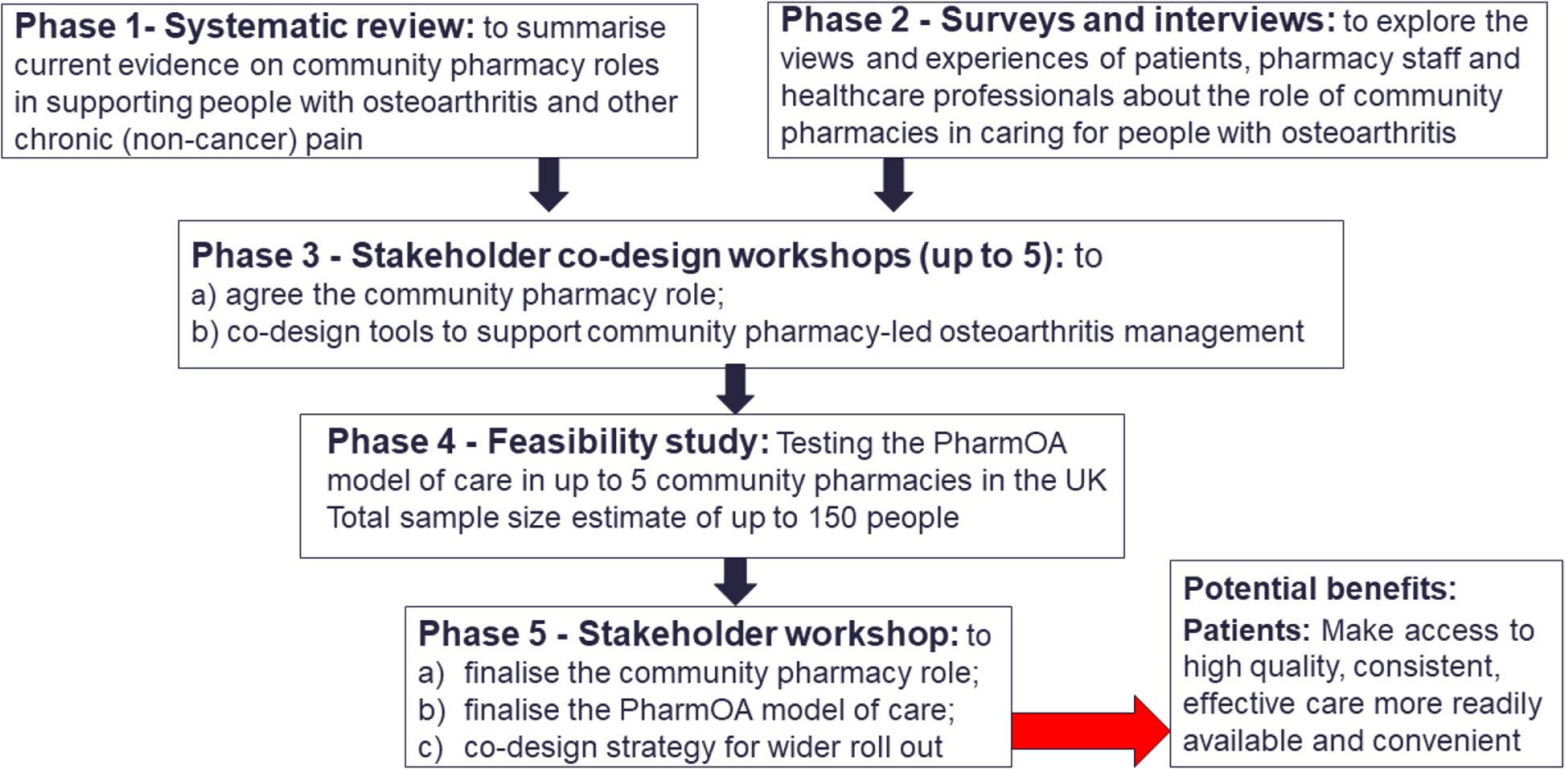
PharmOA study flow

Informed by the Theoretical Domains Framework (TDF), Normalisation Process Theory (NPT), and the Medical Research Council (MRC) framework for developing complex interventions [25–29], we will undertake a five-stage multi-methods study (see Fig. 1) to address the following objectives:

- 1. Summarise currently available evidence on community pharmacy’ roles in supporting adults with osteoarthritis and other chronic (non-cancer) pain (Phase 1; systematic review).
- 2. Explore views and experiences of patients, healthcare professionals, and pharmacy staff about current, and potential extended community pharmacy roles in delivering osteoarthritis care (Phase 2; cross sectional surveys and one-to-one interviews).
- 3. With stakeholders: a) agree on the extended role of community pharmacies in osteoarthritis care; b) co-design a model of service delivery within which the extended roles could be delivered (PharmOA model of service delivery); c) refine existing tools to support community pharmacies to deliver extended osteoarthritis care roles (PharmOA tools) (Phase 3; stakeholder co-design).
- 4. Explore the acceptability and feasibility of the PharmOA model of service delivery (and associated PharmOA tools) to community pharmacy teams when providing care for people with osteoarthritis (Phase 4; feasibility study).
- 5. With stakeholders: a) finalise the PharmOA model of service delivery (and associated PharmOA tools), based on the findings of the feasibility study, and b) if applicable, prioritise recommendations for its wider evaluation and implementation (Phase 5; stakeholder workshop).

Ethical approval will be sought for this study.

### Embedded patient and public involvement

This programme of work arose from, and was shaped by, people with osteoarthritis and has embedded patient and public involvement (PPI) throughout. Our PPI study team member (CW) has contributed to the study design and funding acquisition. She will also support systematic review analysis, survey and interview tool development, and evaluation of the PharmOA model of service delivery (and associated PharmOA tools) and will help shape recommendations from this research and co-produce dissemination materials. PPI representatives will also be involved in stakeholder workshops within Phases three and five of the study. All PPI contributors to the study will be reimbursed according to the National Institute for Health and Care Research (NIHR) guidance [24].

### Underpinning conceptual frameworks

Overall, the PharmOA programme of work is underpinned by the MRC framework for developing complex interventions [29]. Specifically, the TDF will be used to guide the development of the PharmOA model of service delivery (surveys, interviews and stakeholder co-design). The TDF is a framework for understanding and addressing behaviour change. It includes 14 domains that are considered to influence behaviour and behaviour change (i.e., knowledge, skills, social/professional role and identity, beliefs about capabilities, optimism, beliefs about consequences, reinforcement, intentions, goals, memory, attention and decision processes, environmental context and resources, social influences, emotions, and behavioural regulation) [25]. The NPT specifically addresses factors needed for successful implementation and integration of interventions into practice [26]. Given the objective to improve access to high quality osteoarthritis care in the community, the NPT will also be drawn upon to conceptualise the feasibility of the PharmOA tools considering future potential implementation and integration into the primary healthcare system (feasibility study). The TDF and NPT will both inform testing of the PharmOA model of service delivery (and associated PharmOA tools) and the development of strategic recommendations for the future implementation of community pharmacy model of service delivery for osteoarthritis, consistent with national evidence-based guidelines.

### Phase 1: systematic review

A systematic review will be undertaken to explore the role of community pharmacies in supporting adults with osteoarthritis and other chronic (non-malignant) pain (PROSPERO ID: CRD42022320613). Using a comprehensive search strategy, nine databases (including MEDLINE, EMBASE, CINAHL, PsycINFO, Cochrane Database of Systematic Reviews-CDSR and Web of Science) will be searched to identify relevant primary studies. To be eligible for inclusion, empirical studies (including qualitative studies, surveys, cohort studies, mixed methods studies, randomised/quasi randomised controlled trials (RCTs), pilot and/or feasibility studies, non-randomised intervention studies) must have investigated osteoarthritis or other chronic (non-cancer) pain care for adults, delivered by pharmacists or any member of the pharmacy team in community settings. Studies must have reported at least one clinical outcome of interest (e.g., pain intensity, physical functioning, quality of life) or adverse events, or acceptability of interventions, attitudes, beliefs, and behaviours or cost-effectiveness analyses. There will be no exclusion based on language or country of origin. The review process will be managed using Covidence software [30]. Following a pilot screening phase to validate application of eligibility criteria, titles and abstracts will be single screened, and two reviewers will independently undertake full-text reviews. Specifically, titles and abstracts will be screened by a single reviewer (AC) with 10% sample cross-check between two other reviewers (MH, NO). Eligible full texts will be screened by two independent reviewers (AC and JS). Disagreements will be resolved by the independent adjudication of a third reviewer (MH, OB, SW, NO, AC) or in team discussion. Quality assessment will be conducted using the Critical Appraisal Skills Programme (CASP https://casp-uk.net/casp-tools-checklists/) set of appraisal checklists by two independent reviewers (with a third reviewer resolving disagreements). Quality assessments will inform interpretation but not exclusion of studies. Data extraction will be undertaken using a modified Covidence Extraction 2.0 data extraction template (tailored to the study aims and piloted a priori). Data items will include study characteristics (study design, country of origin, participant information), details of interventions (informed by Tidier checklist) [31], outcomes of interest, and key findings. A narrative synthesis of evidence will be conducted, including identification of gaps in evidence and areas of uncertainty. Findings of the systematic review which will be reported in line with PRISMA guidance [32] and will be used to inform subsequent study phases (Fig. 1 PharmOA overall study scheme).

### Phase 2: survey and interview study

#### Phase 2a: surveys

Three cross-sectional surveys will be completed with:

a. Patients aged 45 years or older registered at a general practice in England who have consulted for osteoarthritis or joint pain within the last 2 years.

b. Health care professionals (including, but not limited to, doctors, nurses, physiotherapists, other first contact practitioners) practising in primary care or the community in the UK who have seen at least one person aged 45 years and over with joint pain and osteoarthritis in the last 6 months.

c. Pharmacy team members working in primary care or community settings (e.g. pharmacies situated in high street locations, neighbourhood centres, or supermarkets).

The full eligibility criteria for each survey are described in Table 1.

**Table 1:** PharmOA study population – eligibility criteria

	Inclusion Criteria	Exclusion Criteria
Patient	The target population are adults aged 45 years and over with joint pain and osteoarthritis Registered at one of up to six general practices within the North East or West Midlands in the UK Recorded osteoarthritis or joint pain consultation in the last two years Able to provide informed consent Willing to participate	Anyone under 45 years old Registered at any other than the 6 selected UK general practices Those over 45 who have not consulted for joint pain or osteoarthritis in the past 2 years Vulnerable individuals (e.g., in palliative phase of care, unstable mental health disorders)
Community pharmacists	Registered pharmacists or a member of the pharmacist team working in primary care or community settings Practicing in the UK Able to provide informed consent Willing to participate ^a^ By community pharmacies, we mean teams of pharmacies (e.g., pharmacists, dispensers, pharmacy assistants, pharmacy technicians) within the heart of the community; usually situated in high street locations, in neighbourhood centres, located within non-dispensing GP Practices buildings and in supermarkets	Community pharmacy staff working in secondary care or other non-primary care settings Student Community pharmacists Community pharmacy staff not practicing in the UK
Healthcare professionals	Any qualified healthcare professional working in community or primary care settings (including but not limited to GPs, nurse practitioners, practice nurses, physician associates, first contact practitioners, physiotherapists, occupational therapists) Treated at least one patient with osteoarthritis (defined as adults aged 45 years and over with joint pain) in the last 6 months Practicing in the UK Willing to participate and able to provide informed consent	Healthcare professionals not working in primary care or community settings Healthcare professionals that have not treated at least one patient with osteoarthritis in the last 6 months Unqualified/student/assistant Healthcare professionals Pharmacists (these individuals will complete a different survey) Healthcare professionals not practicing in the UK

^a^ For the interview study, all participants (patients, Community pharmacists, Healthcare professionals) must have:

• Returned the survey

• Provided consent for further contact

• Access to a telephone, or computer with internet for accessing the virtual interview platform Microsoft teams

### Sampling and survey processes

#### Patient survey

Eligible patients will be identified through computerised electronic record screening of participating general practice databases (*n* = up to 6, located in both high and low deprivation areas in the North East and West Midlands regions in England) based on joint pain related diagnostic or symptomatic SNOMED Codes (also previously known as Read codes). A single survey sent from the patients registered general practice will be posted to eligible patients (containing a cover letter, participant information leaflet, paper-based questionnaire including a consent form for further contact and a pre-paid return envelope).

#### Community pharmacy

Pharmacists and pharmacy team members working in primary care and community settings in the UK will be invited to complete an online survey using broad based advertisements via professional organisations, the NIHR Clinical Research Network, and social media (e.g., Twitter); email invitations to participate via publicly available email addresses (e.g., from clinical commissioning groups (CCGs) websites), professional contacts of study team members, and snowballing. Study advertisements and email invitations will include a link to the community pharmacy online survey that will include participant information, the questionnaire, and a consent form for further contact.

#### Healthcare professionals

Healthcare professionals from across the UK will be invited to complete an online survey commenced via the same methods outlined for the community pharmacy survey.

### Sample size

#### Patients

Informed by the response to first-round mailings of similar research and surveys [33–37], assuming a conservative 20% response, it is expected that a single mail out to approximately 2000 patients will be sufficient to achieve the desired sample size of 400 participants (patients). This will enable estimation of any proportion of interest with a precision of ± 5% or better, based on a 95% confidence interval [38, 39].

#### Community pharmacy and healthcare professionals

Based on previous similar surveys [37, 40–43] we expect between 100 and 400 responses from healthcare professionals and pharmacy staff. This will enable estimation of any proportion of interest with a precision of between ± 10% and ± 5%, on a 95% confidence interval [37–39].

### Survey instruments

The questionnaires for the three surveys will be adapted from previous instruments [9–11, 44]. As well as drawing on findings from previous literature, team expertise (academic research and clinical practice), and PPI input, questions will be theoretically informed by the TDF [45, 46] and the NICE osteoarthritis guidelines [3]. For example, our survey instruments assess behaviour and behavioural change constructs of the TDF such as knowledge of the NICE OA guidelines, perceived skills, professional role and identity relevant to an extended Pharmacy role in OA care, beliefs about capabilities, decision processes, contexts and resources that may influence real world implementation of an extended pharmacy role for OA care. Each survey instrument will be refined as appropriate following testing in a pre-pilot study with three eligible potential participants, respectively. Using questions with predominantly closed-option responses (with an option of ‘other’ and space to provide other relevant information), the questionnaires will capture descriptive data on: demographics, experience of osteoarthritis care (patients) and care provision (pharmacy staff and healthcare professionals), views about the current and potential extended role of community pharmacies in the management of osteoarthritis, and perceived barriers and facilitators to the potential delivery of this extended role.

At the end of each questionnaire, a final section will seek respondents’ consent for further contact. Those who agree to further contact will form the sampling frame for the interview study.

### Data analysis

The Health Survey database held by Keele University will be used for data entry of responses from the patient survey. (Cross checks (~ 1 in 5) will be carried out to ensure reliability and quality assessment of data entry.

Descriptive statistical analyses (numbers, percentages, measures of central tendency, standard deviations, medians, interquartile range as appropriate) will be undertaken to summarise the findings. Quantitative estimates will be presented with 95% confidence intervals as appropriate.

Further descriptive exploratory analysis will be conducted to investigate whether any differences exist between the three sampled groups in relation to attitudes and beliefs about, and potential barriers to delivering, an extended community pharmacy role in managing osteoarthritis. In addition, if numbers allow, we will stratify key results within each survey by the following factors: age, gender, ethnicity, comorbidity, duration, site, and severity of osteoarthritis symptoms (patient survey), context of care, professional background/experience and geographical region (community pharmacy and healthcare professional survey). Statistical tests (e.g., Chi-square tests/t-tests/Analysis of variance) will only be used in an exploratory context to help guide where large differences may exist in the data and where further investigation of the finding may be beneficial in the later stages of the study.

### Phase 2b: patients, healthcare professional and community pharmacy interviews

**Fig. 2:**
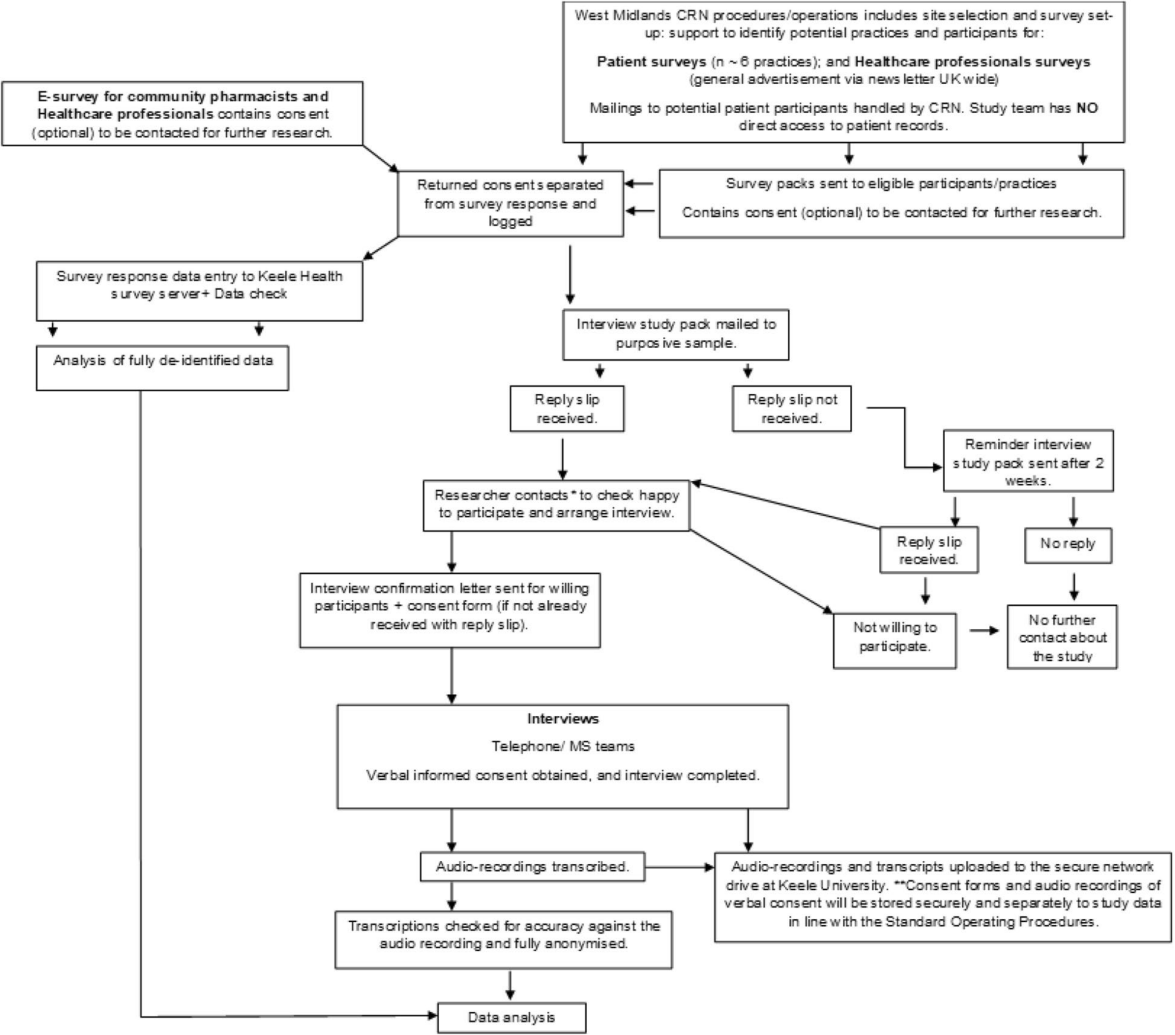
Interviews and surveys participant consent/data collection procedures

Within each group (patients, community pharmacy, healthcare professionals) one-to-one semi-structured interviews will be completed with up to 20 questionnaire respondents who provide consent for further contact (see Fig. 2 for the recruitment, informed consent, and data collection procedures). Where numbers allow, interviewees will be purposively selected to represent the broadest range of characteristics including age, gender, ethnicity, duration/severity of symptoms (patients), role (healthcare professionals – e.g., GPs, nurses, physiotherapists), context of work (pharmacies), duration of professional experience (healthcare professionals, community pharmacies) and geographical area. Data collection in each group will cease once all available participants have been interviewed, or sooner if thematic saturation is reached [47–49].

Following obtaining informed consent, interviews will be conducted remotely via Microsoft Teams, or over the telephone. A topic guide will be followed to gain more depth of understanding about participants’ current experiences of care, the potential extended role of community pharmacy in osteoarthritis care, and perceived potential barriers and facilitators to its delivery. Open questions will allow participants to discuss anything else perceived to be relevant and important. All interviews will be recorded, fully transcribed, checked for accuracy and fully anonymised prior to analysis.

### Analysis

Thematic analysis of verbatim transcripts will occur alongside data collection so that emerging themes can be explored in subsequent interviews [47]. A two-stage framework will be adopted. Initial inductive thematic analysis will identify themes within the data which will later be mapped onto domains within the TDF [45–48]. First, each interview transcript will be initially read and re-read to identify and code discrete parts of the data that represent particular concepts [48, 49]. Using principles of constant comparison, data will be closely examined for similarities and differences. Data representing similar concepts will be grouped into themes [48, 49]. Emerging codes and themes will be discussed and agreed with the research team members (including PPI) and applied to the dataset with ongoing refinement as needed. Inductive analysis will precede deductive analysis and mapping of themes to the TDF. This layered approach enables a rich interpretative analysis to be completed as emergent issues are identified ahead of making sense of data according to theoretical constructs. Furthermore, mapping of themes to the TDF will be undertaken by researchers from different professional backgrounds, to improve trustworthiness of the analysis.

### Phase 3: stakeholder co-design workshops

#### Participants

A panel of stakeholders (*n* = up to 50) will be convened, including patients, members of community pharmacy teams, healthcare professionals (including GPs, nurse practitioners, physician associates, physiotherapists, urgent care practitioners, patients and/or their carers), and relevant third sector representatives. They will be identified through clinical and research networks, authorship of relevant publications, survey and interview participants (who have agreed to further contact, and personal networks of the study team. Informed consent will be taken for each panel member prior to the workshops commencing.

#### Procedure

Up to 5 workshops will be convened, in line with core principles of co-design, including power sharing, and participatory approaches [50, 51]. They will be facilitated by experienced PharmOA researchers in co-design and intervention development. Prior to attending each workshop, panel members will be sent up to date information about the study progress and findings. All workshops will last approximately 2 h and will be held remotely (via Microsoft Teams) so that geographical distance is not a barrier to participation. Patient panel members will be offered dedicated support (including pre workshop demo, technical information guide, on the day 1:1 support via telephone) as needed to address technical issues that may act as barriers to active participation in online meetings. All group discussions will be audio-recorded and further data will be captured through field-notes taken during the workshops, ensuring that all decision-making processes are captured, including discussions leading up to final agreements.

The first one to two workshops will be used to gain consensus on the optimal extended role of community pharmacies in delivering care for people with osteoarthritis. This will be achieved via a modified nominal group technique (NGT) including presenting information, silent ideas generation, group discussions rating of consensus items, and further discussions where appropriate. Post-workshop(s), the PharmOA study team will consider how the agreed extended role of community pharmacy could be delivered in practice (based on the findings from the systematic review, surveys and interviews), and the tools that could be used to support its implementation (PharmOA model of service delivery and PharmOA tools).

As key stakeholders, community pharmacy staff will be invited to participate in the next two workshops, where the draft PharmOA model of service delivery and PharmOA tools will be further developed and refined using co-design techniques. This will occur through semi-structured group discussions, considering different patient profiles, and different care pathways which may be applicable to the care of osteoarthritis in real world practice and core management approaches outlined by the NICE osteoarthritis guidelines [3]. The output of the workshops will be refined content and design features of the PharmOA model of service delivery and PharmOA tools.

All stakeholders will be invited to attend the next workshop where the PharmOA model of service delivery and associated PharmOA tools will be presented and discussed. Final refinements will be made ready for testing Phase 4 of the study (see below).

### Phase 4: feasibility study

Specific objectives of the feasibility study are to:

- 1. examine if and how the PharmOA model of service delivery (and associated PharmOA tools) is used in up to 5 community pharmacies in England for up to 150 people aged 45 years and over with joint pain and osteoarthritis.
- 2. explore community pharmacy staff experiences of, and views about using the PharmOA model of service delivery and PharmOA tools to support care provision for people aged 45 years and over with joint pain and osteoarthritis.
- 3. explore the barriers and facilitators to using the PharmOA model of service delivery and PharmOA tools.

**Fig. 3:**
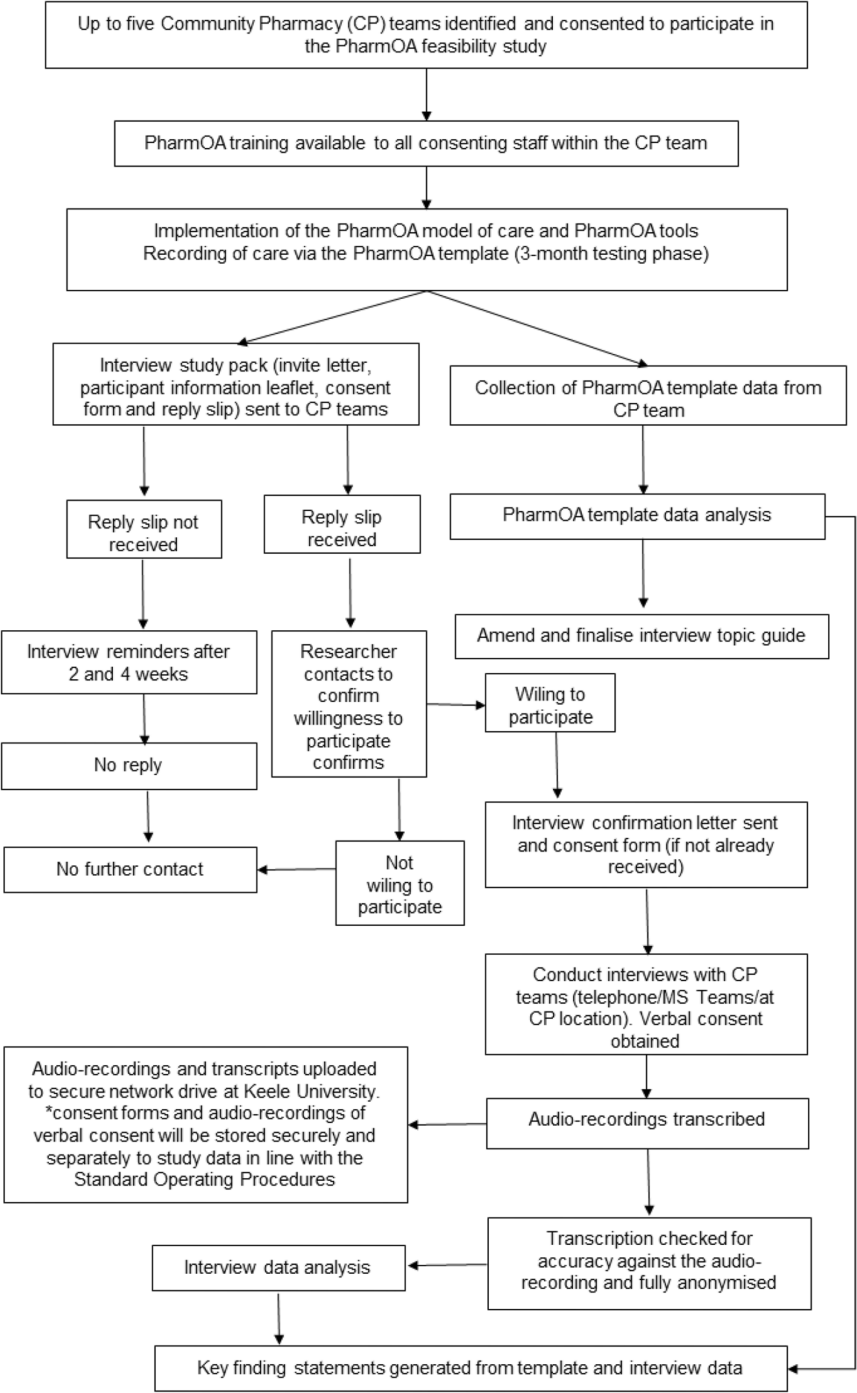
Feasibility study flow chart and process

Up to five community pharmacies in England, will be recruited via the study team’s professional networks (see Fig. 3 for the feasibility study flow). All members of staff at the community pharmacy will be invited to participate in the study through an invitation pack. This will include an invitation letter, information leaflet, brief questionnaire, consent form and pre-paid envelope.

### Training

Following obtaining of informed consent, community pharmacy teams will complete the necessary online training (short video modules; approximately 90 min in total). Within the training, the Health Behaviour Change Competency Framework [52] will be used to describe the competences required to deliver the lifestyle behaviour change and self-management components of the PharmOA model of service delivery. Community pharmacy teams will then be asked to use the PharmOA model of service delivery supported by the associated PharmOA tools with up to 150 people aged 45 years and over with joint pain and osteoarthritis (in their hands, hip, knees, or feet) over a period of 3 months. Community pharmacy teams will be asked to use the PharmOA model of service delivery supported by the associated PharmOA tools over a period of 3 months followed by “formal evaluation”. Pharmacies will be supported to participate in the feasibility study through payment to compensate for staff time to complete the online training, deliver the intervention (PharmOA model of service delivery) and attend an interview. Each Pharmacy will also be provided with a study training manual and all the materials needed (e.g., Versus Arthritis leaflets, posters).

Data for this feasibility study will be collected via:

- a baseline questionnaire completed by participating community pharmacy staff.
- a PharmOA care record template to capture care provision, completed by community pharmacy teams
- Semi-structured interviews with participating community pharmacy staff.

### Baseline questionnaire

All consenting members of the community pharmacy teams within participating pharmacies will be asked to complete a brief baseline questionnaire. Demographic information including role, gender, ethnicity, years in practice, training in osteoarthritis, knowledge of osteoarthritis care and management, and their experience and frequency of supporting and delivering care to people with osteoarthritis will be collected. In addition, data will be collected on brief characteristics of the community pharmacy (e.g., location and size of practice, team composition and organisation of care). This information will enable description of participating pharmacy teams and will enable contextual interpretation of facilitators and barriers to successful future implementation of the PharmOA model of service delivery.

### PharmOA care record template

All participating members of the community pharmacy teams will be asked to complete a care record template when they provide care for anybody aged 45 years and over with joint pain and osteoarthritis using the PharmOA model of service delivery and PharmOA tools. Descriptive statistical analysis (numbers, percentages, means and standard deviations as appropriate) will be used to summarise the use of the PharmOA approach and PharmOA tools and the care provided for osteoarthritis throughout the 3-month feasibility testing phase. With a sample size of up to 150 people we will be able to estimate any proportion of interest with a precision of ± 9% on a 95% confidence interval [37–39].

### Qualitative component: semi‐structured interviews

At the end of the 3-month feasibility study phase, consenting members of participating community pharmacy teams will be invited to a one-to-one semi‐structured interview to explore:

- 1) their experiences of, and views about, the PharmOA training programme.
- 2) their experiences of, and views about, the feasibility and acceptability of the PharmOA. model of service delivery and PharmOA tools.
- 3) what factors helped or hindered use and delivery of the PharmOA model of service delivery and associate PharmOA tools.

Interviews will either take place over the telephone, virtually via Microsoft Teams, or face-to face either at Keele University or in the community pharmacy. A topic guide will be used to stimulate dialogue, the structure and content of which will be developed by the study team in conjunction with PPI input, and theoretically informed by the TDF and NPT [27, 28]. Open questions at the end of the interviews will allow participants to discuss anything else they think to be relevant and important, and further exploration of any unanticipated issues raised by participants during the interview. Thematic data analysis will be used following the same procedures outlined in the Phase 2 (interview) study above.

Findings from both quantitative and qualitative components of this feasibility study will be analysed and used to inform Phase 5 of the study.

### Phase 5: final stakeholder workshop

A final stakeholder group will be convened to discuss the findings of the feasibility study and to refine (if necessary) and finalise the PharmOA model of service delivery and associated PharmOA tools. Another important focus is to define and prioritise recommendations (through individual panel member suggestions and collective collation) for wider evaluation and implementation. For example, where appropriate, recommendations may include:

i) Further testing to ascertain effectiveness of the PharmOA model of service delivery and address potential implementation challenges.
ii) Further training plan for supporting community pharmacy teams to continue to deliver evidenced-based osteoarthritis care.
iii) Public awareness raising strategy for wider dissemination of education materials for osteoarthritis and awareness of extended community pharmacy role in managing osteoarthritis.
iv) Best practice/policy briefs for pragmatic implementation into local contexts and care commissioning.

Stakeholder group discussions will be audio-recorded and further data will be captured through field-notes taken during the workshops. This will ensure that all decision-making processes are captured, including discussions leading to final recommendations.

## Discussion

Living with LTCs like osteoarthritis can be challenging [53]. As part of the National Health Service Long-Term Plan, supported self-management has been proposed as a good way to reduce the impact of long-term conditions [19]. Given the unique position of the community pharmacy as a readily accessible first point of contact for people with LTCs, supporting community pharmacies in delivering more holistic, evidence-based care for osteoarthritis is imperative. The PharmOA study may provide the opportunity to effectively support and empower people to self-manage and live well with osteoarthritis.

Overall expected outputs for this study are:

- New knowledge about current community pharmacy care for osteoarthritis
- An agreed extended community pharmacy role for the management of osteoarthritis
- A model of service delivery and associated tools within which the extended role can be delivered
- A set of recommendations to be considered for wider future implementation (scale up and scale out) of enhanced support for osteoarthritis care in community settings, including public awareness raising strategy.

The main value is trying to standardise and improve care for people with osteoarthritis wherever they seek help from. Our multi-methods design including quantitative and qualitative data has added richness and depth of understanding to the development of, the facilitators and barriers to the provision of quality care for people with osteoarthritis. Incorporating learning from previous work with GPs, and nurses in primary care settings [54], we are taking a pragmatic approach to the conduct of the PharmOA study, ensuring processes, delivery and data capture are closely aligned with real world practice. Our co-design approach with relevant stakeholders will also ensure important issues are addressed at the development phase considering potential scale up and scale out implementation in future. Through embedded PPI activities and partnerships, this project will contribute to the growing body of evidence on ways of extending PPI beyond research and dissemination activities [55] to mobilising evidence-based guideline recommendations into real world benefits.

## Data Availability

Relevant materials to this study protocol are included in this published article.

## Abbreviations

CRN: Clinical research network
HCP: Healthcare professional
LTC: Long term condition
GP: General practitioner
TDF: Theoretical domains framework
NPT: Normalisation process theory
NIHR: National institute for health and care research
PPI: Patient and public involvement
